# The development of an online measure of therapist competence^☆^

**DOI:** 10.1016/j.brat.2014.11.007

**Published:** 2015-01

**Authors:** Zafra Cooper, Helen Doll, Suzanne Bailey-Straebler, Dorothea Kluczniok, Rebecca Murphy, Marianne E. O'Connor, Christopher G. Fairburn

**Affiliations:** a Oxford University, Department of Psychiatry, UK; b ICON Patient Reported Outcomes, Oxford, UK

**Keywords:** Therapist competence, CBT-E, Eating disorders, Online measure, Rasch analysis

## Abstract

The topic of therapist training has been relatively neglected in the research literature. Similarly, the related issue of the measurement of the outcome of training, especially therapist competence, has been largely overlooked. Data supporting the effectiveness of various methods of clinician training and those providing estimates of the level of competence achieved by clinicians are scarce. Validated scalable methods for the measurement of clinician outcomes such as competence are required to evaluate both existing and new methods of training. This study focuses on the development and testing of an online measure (eMeasure) to assess therapists' applied knowledge of Enhanced Cognitive Behaviour Therapy (CBT-E), a transdiagnostic evidence-supported treatment for the full range of eating disorders. The eMeasure meets the stringent requirements of the Rasch model and has three equivalent versions making it suitable for repeat testing of trainees in outcome studies. Preliminary best cut points to distinguish between those who are competent and those who are not are identified. While the present work focused on CBT-E, the method described may be used to develop and test other measures relating to therapist competence.

## Highlights

• The development and testing of an online measure of CBT-E competence is described.
• Uses Rasch model to estimate latent ability from item responses.
• Three equivalent versions of the measure for use in outcome and follow up studies.
• Describes a method for developing scalable measures of therapist competence.
• Method may be applied to devising competence measures for other treatments.

The topic of therapist training has been relatively neglected in the research literature. Similarly, the related issue of the measurement of the outcome of training, especially therapist competence, has been largely overlooked (Fairburn & Cooper, 2011).

Data supporting the effectiveness of various methods of clinician training and especially those providing estimates of the level of competence achieved by clinicians are relatively scarce (McHugh & Barlow, 2010) and there are consistent calls for more rigorous research methods to measure such outcomes. In particular, the need for validated scales for the measurement of clinician outcomes such as competence has been highlighted (Beidas & Kendall, 2010; Rakovshik & McManus, 2010; Shafran et al., 2009). The lack of consistency in measurement in this area has contributed to making existing training research difficult to interpret (Herschell, Kolko, Baumann, & Davis, 2010; McHugh & Barlow, 2012).

The present study is part of a larger programme of work designed to develop scalable methods of training therapists in evidence-supported psychological treatments. Valid procedures for evaluating training outcome are essential prerequisites for testing these new methods. These need to be able to assess therapist competence to implement a specific treatment by testing therapists' theoretical knowledge of the treatment as well as their applied knowledge of how and when to use it and its various strategies and procedures. In addition, therapists' ability to apply this knowledge in practice needs to be assessed. Clearly, the project of scalable training also requires that the outcome of training is measured in a scalable way. In this report we focus on describing the development and testing of an online measure (eMeasure) to assess therapists' applied knowledge of Enhanced Cognitive Behaviour Therapy (CBT-E) (Fairburn, 2008), a transdiagnostic evidence-supported treatment for the full range of eating disorders (Dalle Grave, Calugi, Doll, & Fairburn, 2013; Fairburn et al., 2009, 2013; Poulsen et al., 2014). While the content of the measure is, of course, specific to CBT-E, the general principles involved in its development and testing apply generally and are also a focus of this report.

## Method

### Design

This study was conducted in two phases. In the first phase a bank of items was developed to provide the potential content of the eMeasure. In the second phase, the items were evaluated in order to obtain a pool of calibrated items fitting the Rasch model (see below) and provisional best cut points for competence were established. Clinician trainees provided responses to one of three versions of the measure with each version comprising a subset of the total items developed. For later comparison purposes these tests were linked by a number of common or anchor items. Ethical approval was obtained from Oxford University Central Research Ethics committee.

### Phase one – development of items

#### Content definition

The first step was to identify the full range of knowledge required by clinicians to implement CBT-E. This included theoretical knowledge (knowing *that*) about the treatment including its indications, contraindications, stages and strategies; and applied knowledge (knowing *how*) about how to implement the treatment in given clinical situations. To ensure adequate coverage and sampling of the potential content, we developed a “blueprint”. Blueprints systematically seek to link the definition of the content to be learned with the specific items that are covered by the assessment measure (Bridge, Musial, Frank, Roe, & Sawilowsky, 2003; Coderre, Woloschuk, & McLaughlin, 2009). This involved a two stage process. Using the evidence-based treatment guide as our source (Fairburn, 2008), we obtained agreement among three treatment experts (ZC, RM and SBS) concerning the essential elements in each of the stages of treatment. It was decided not to involve CF in the development of the content of the eMeasure as he was developing the CBT-E training programme, and we wished to avoid the risk of “teaching to the test”. The blueprint (see Table 1) was drawn up in accordance with a number of general considerations, applied in this case to CBT-E. We decided that the greatest proportion of items should cover the two major stages in treatment (Stages One and Three) and that these two stages should have roughly equal weight represented by roughly equal numbers of items. Stage Two, the transitional review and planning stage, was included within Stage Three. During training, most teaching time is spent on Stages One and Three. While Preparation for treatment and Stage Four are also essential elements of treatment, the knowledge and skill required to implement them is more generic and clinicians require less training to implement these well.

#### Item generation

Two types of multiple choice question were selected as best suited for assessing the content we wished to address: the one-best-answer format requiring test takers to select a single best response and a variation of this format that asks the test taker to choose a specified number of options. In line with general recommendations for such testing (Case & Swanson, 2002), we decided that a substantial proportion of the questions would assess applied knowledge, covering the application of treatment strategies and problem solving clinical issues and common difficulties (see Table 1). The majority of the questions would therefore involve a clinical vignette or the presentation of a sample patient record typical of that used in treatment. Examples of a theoretical knowledge question, questions involving a clinical vignette and one involving a patient record are provided in supplementary material accompanying this report (available online). The examples supplied are of items that were not eventually included in any of the final versions of the test. While these items have less sound psychometric properties than those retained, they illustrate the nature and structure of the test items.

Multiple items were written for each topic on the blueprint (ZC) while reviewing and editing was done by a group of CBT-E experts with the help of an independent expert^1^. Distractors or incorrect options were designed to be plausible, grammatically correct and of the same relative length as the correct answer. In accordance with item writing guidelines (Haladyna, 2004), commonly observed or typical errors (in this case those made by trainee CBT-E therapists) were used to generate plausible but incorrect distractor items. A total score was calculated as the sum of all correct responses. As there was no a priori reason for some items to have greater weight than others it was decided to use a binary scoring system with each item having equal contribution to the total score.

#### Initial item pool

Our goal was to produce at least three versions of the measure with equivalent content. After an informal ‘tryout’ of items with a sample of 5 potential users 67 items were selected for further evaluation from our initial pool of 75 items. The remaining 8 items were eliminated during this review process for a number of reasons: potential users reported ambiguity in the wording; it became apparent that there were alternative correct answers or, on further inspection, items violated item writing guidelines in some other way (Case & Swanson, 2002).

The 67 items were subdivided to create three versions of the eMeasure, each version comprising 19 unique items and 9 common (anchor) ones. The one remaining item was not used at this stage but was retained for further trials. To ensure approximately equivalent content, the 19 unique items were drawn from sets of similar items covering all the stages of treatment in the proportions dictated by the blueprint. The nine common items also covered all the stages of treatment and occurred in similar positions in each version of the test.

#### Measure administration

The three versions of the eMeasure were administered using software developed for summative online examinations (ExamOnline)^2^ hosted on an Oxford University server. Test takers were able to follow an emailed link and complete the test without needing sophisticated computing skills. Scoring was fully automated.

### Phase two – evaluation of the test items

#### Recruitment of CBT-E trainees

To examine the performance of the test items we recruited a large and heterogeneous sample comprising the full range of clinicians (in terms of their professional background and stage of training) for whom training in CBT-E is relevant. More than 500 (*n* = 520) clinicians, agreed to participate.

By design the clinicians were selected from those at various stages in training: prior to attending a conventional training workshop or prior to embarking on an online form of training; after attending a 2 day training workshop (by CF); and after receiving a course of expert-led clinical supervision in CBT-E. Amongst this latter group 22 clinicians were viewed as clearly competent in CBT-E as judged by expert clinicians and treatment developers (ZC and CF). The judgement was made on the basis of the clinicians' knowledge of the treatment and their clinical work.

The clinicians were asked by CF if they would volunteer to complete the eMeasure in order to help with its development and refinement. Those who agreed were sent an email with separate links to the eMeasure and to an online questionnaire survey which asked for basic demographic data (age, professional background, years of clinical experience). They were assured that their eMeasure scores would remain confidential.

An initial cohort of 112 clinicians (of the 520) was asked to complete one of the three 28 item eMeasures (Tests A, B, and C). They were allowed up to an hour to do so although we estimated that each test could be easily completed within 40 minutes. This was to avoid confounding test takers' performance with the irrelevant difficulty of speed of working. Their responses were used to determine the acceptability and feasibility of the testing method. Total test scores were calculated and tabular inspection of the number of correct responses for each item in relation to mean total test scores was used to identify items which were poorly functioning and which should be removed (Haladyna, 2004). A second cohort comprising the remaining 408 clinicians was asked to complete one of three revised tests (A-27, B-27, C-27).

### Statistical analysis

The Rasch model is a widely used statistical method of estimating latent abilities by studying item responses (Fischer & Molenaar, 1995; Rasch, 1960/1980). It provides a mathematical framework based on the assumption of unidimensionality and local independence against which the test data can be compared. Estimates and standard errors of person ability and item difficulty are calculated on a common equal-interval logit scale. Rasch uses one parameter to estimate person ability (the number of correct responses by a person) and item difficulty (the number of correct responses to an item) to determine the probability of a person *n* succeeding on an item (Rasch, 1960, pp. 62–125).

The Rasch model was fitted within RUMM 2030 version 5.4 for Windows (Andrich, Sheridan, & Luo, 2012) to assess overall item fit to a unidimensional model as well as the fit of individual items and persons. Data from all 66 items (including those excluded from the second testing stage, see above) were included in these analyses. Decisions on item removal were made with reference to the degree of any misfit (with statistical significance for overall misfit taken at *p* < 0.05 and for individual items taken at *p* < 0.01 because of multiple testing) and the extent to which their removal would affect subject coverage of each test. Individual items significantly misfitting (*p* < 0.01) were first excluded in a stepwise and cumulative approach, excluding all items found first to have significant misfit at *p* < 0.01 and then excluding items subsequently found to misfit (*p* < 0.01) after excluding this first set of items. This process continued until there was no overall misfit (*p* < 0.05) and no individual item misfit at *p* < 0.01. The completeness of coverage of each test was then examined, replacing items with least significant misfit as necessary. Finally test scores were equated, using the common anchor items, so as to enable identification of the score on each test which mapped onto the same degree of clinician ability (person ‘location’).

To establish a provisional cut point to distinguish those clinicians independently classed as competent from the rest of the sample, data from the three revised test sets of varying item length (S-22, S-24, and S-25; see below) were fitted in RUMM and the resulting person locations (i.e. abilities) across all three versions of the tests (A, B, C) were extracted. These person locations (i.e. person abilities in terms of applied knowledge) were used rather than the total test scores themselves in the Receiver Operator Characteristic (ROC) analyses. Non-parametric estimation was used to determine the ‘best cut point’ (BCP) from the values of sensitivity and specificity calculated at increasing test score cut-points. Equal weight was given to sensitivity and specificity. These ability estimates were then linked to test scores within RUMM, using the test equating features, to give a test score cut point which could be used as a provisional indicator of competence.

Data are presented throughout as *N* (with %) or mean (with standard deviation, SD) depending on their distribution. Chi-squared tests, with testing for linear trend where appropriate, were used to identify associations between the demographic variables.

## Results

### Test completion

451 (86.7%) of the 520 clinicians contacted and asked to complete a test did so, with 87 (77.7%) of the first cohort of 112 clinicians completing one of the initial 28 item tests and 364 of the remaining 408 clinicians (89.2%) completing one of the three revised tests. Overall, 154 clinicians completed a version of Test A, 154 a version of Test B and 143 a version of Test C.

### Test revision

The initial field testing on the first cohort of 87 clinicians confirmed the acceptability and feasibility of the testing method. Tabular inspection of the data supported the removal of three of the unique items because they either failed to discriminate between the mean total scores of those choosing the correct answer and those choosing the incorrect answer or discriminated in the wrong direction. A further item was removed because as it was not answered correctly by anyone.

After removal of the four poorly functioning items and the replacement of the item not answered correctly by anyone with the previously unused item, three revised 27 item tests were created (A-27, B-27, C-27), comprising 18 unique items and 9 common items.

### Characteristics of the CBT-E trainees

329 of the 451 (72.9%) clinicians who completed the e-Measure provided background demographic data. Their mean (SD) age was 42.8 (9.8) years and the majority (86.2%) were female. Their mean (SD) number of years of clinical experience was 12.8 (9.0) with their professional backgrounds being as follows: clinical psychologists (30.7%), nurse or nurse therapists (19.8%), social workers (8.8%), eating disorder therapists (7.0%) and psychiatrists (4.9%). Around 30% (28.9%) came from a variety of other professional groups. Slightly more than half (53.3%) reported that they encountered patients with eating disorders “often”, with approximately half (49.4%) treating such patients “often”.

### Rasch analysis

#### Unidimensionality and item reduction

The full set of 66 items (28 items per test, showed overall misfit to a unidimensional model (chi-square = 595.1, df = 396, *p* < 0.000001)). The one item that had not been answered correctly by any clinician was excluded by RUMM. Seven individual items showed significant misfit (*p* < 0.01).

Stepwise exclusion of misfitting items (see above) resulted in the removal of 12 items (3 of these being anchor items) including the 3 already identified as performing badly and the one item answered incorrectly by all clinicians. The remaining 54 items showed no significant overall misfit (chi-square = 311.0, df = 324, *p* = 0.688). As can be seen in Table 2, this process resulted in a set of 3 individual tests with 22 items each (S-22), of which 6 were common or anchor items and the remaining 16 were unique items, with no individual item misfitting at *p* < 0.01.

**Fig. 1:**
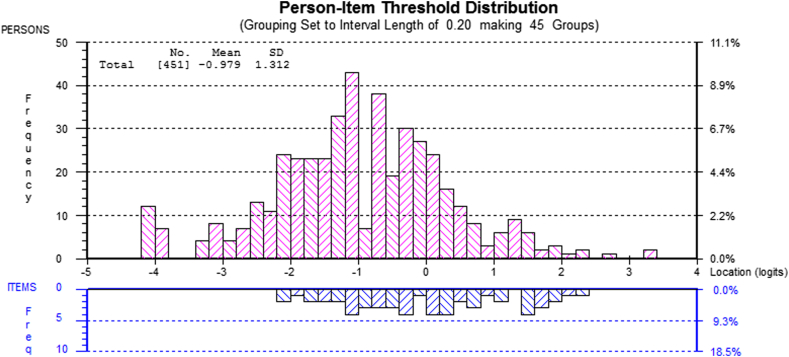
Person–item mapping for reduced pool of 54 items.

Fig. 1 shows the person–item distribution for the reduced pool of 54 items. With the items centred by RUMM at a difficulty of zero, person ability is located at a mean of −0.979 (SD = 1.312). This indicates that, as expected with this mixed population (many of whom were tested before training), in terms of the underlying construct (CBT-E applied knowledge), the items have scope to identify a greater ability on the knowledge measure than that shown by persons in this population and thus to identify increased knowledge as a result of training.

**Fig. 2:**
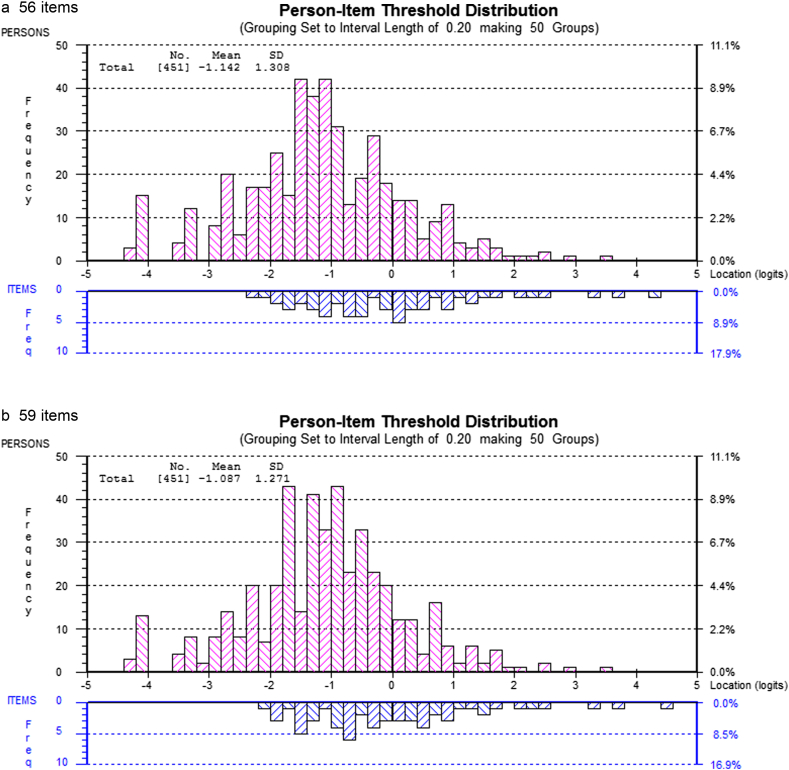
Person–item mapping for item pools containing 56^a^ and 59^b^ items.

Inspection of the 12 excluded items revealed that the loss of these items did not affect the relative proportion of items covering the various stages of treatment as specified in the blueprint. However, two common items covering important content about the treatment structure and three vignette style questions covering the implementation of key interventions in Stage One and Three had been removed. We judged that the removal of these items might compromise the possibility of adequately sampling knowledge of treatment content and so these five items were re-introduced in two separate steps. First the two common (anchor) items were re-introduced so that the total number of items increased to 56 and the set of individual tests each contained 24 items (S-24, each with 16 unique items and 8 common items) and then a further 3 items were introduced covering key clinical interventions increasing the item pool to 59 with the set of individual tests each containing 25 items (S-25, each with 17 unique items and 8 common items). The results of these two new solutions (S-24 and S-25) in addition to the initial S-22 in terms of both overall fit and item fit can also be seen in Table 2. The effect of adding the further items, producing the 56 and 59 item solutions, on the person–item distributions can be seen in Fig. 2. As can be seen in both cases the person–item mapping is slightly less good than the 54 item solution with the items being marginally harder relative to the population.

#### Establishing a cut point score for therapist competence

On ROC analysis, the best cut points (BCPs) overall, differentiating the ‘competent’ from the other ‘non-competent’ clinicians, were a location (ability level) of 0.27 for S-22 (AUC = 0.964, sens = 0.909, spec = 0.881), a location of 0.23 for S-24 (AUC = 0.972, sens = 0.909, spec = 0.907) and a location of 0.13 for S-25 (AUC = 0.969, sens = 0.909, spec = 0.895). As can been seen, the 24 item set (S-24) had a slightly higher AUC (0.972) and slightly better specificity than the other two sets.

**Fig. 3:**
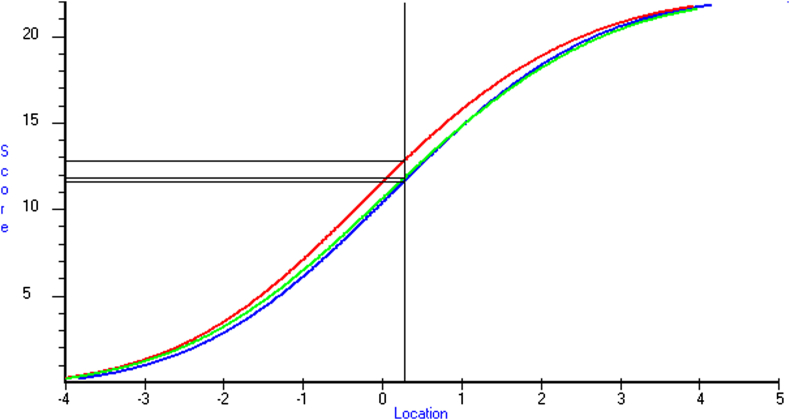
Best cut point for S-22 showing equating of test versions A^1^, B^2^ and C^3^ (^1^Blue = 11.64; ^2^Red = 12.81; ^3^Green = 11.82).

The test curves in Fig. 3 show the equating of the test scores on the three versions (A, B, C) of each test within the S-22 set and indicate the test score (sum of correct responses) that is equivalent to the BCP location. As can be seen, on S-22 the provisional cut point for test B was 13 and for those receiving test A-22 or C-22 it was 12. On S-24 and S-25 the provisional cut point for Test B was 14 and on A-24 and C-24 and A-25 and C-25 it was 13 (not shown).

## Discussion

As we have argued previously (Fairburn & Cooper, 2011), applied knowledge is an essential component of therapist competence. This report describes the development and testing of a brief scalable measure for testing applied knowledge of CBT-E. Three equivalent versions of the measure were developed to allow repeat testing of trainees in follow-up studies. Preliminary best cut points to distinguish between those who are competent and those who are not were also identified. While the present work focused on CBT-E, the method described may be used to develop and test measures relating to therapist competence in other treatments.

The psychometric properties of three sets of tests of varying length are described. The shortest 22 item (in each of the three tests) set of tests conforms most closely to the required assumptions of the Rasch model but it does so at the expense of a losing a broader coverage of the content of CBT-E. Re-introducing items to better cover the content produced (sets with 24 and 25 items in each test) less good model fit. Without further testing and replication on a new sample at least as large as the present one, it would be premature to discard the five items that do not fit as well as the others.

The present study has a number of strengths. There was careful attention to ensuring the validity of the measure by following best practice in test construction as set out in the Standards for Educational and Psychological Testing (American Educational Research Association, American Psychological Association, National Council on Measurement in Education, Joint Committee on Standards for Educational, & Psychological Testing (US), 1999) including blueprinting, training in item writing, independent review of items and initial field testing (Downing, 2006). The sample tested was heterogeneous, covering all those for whom the measure is intended, and relatively large. The data showed good fit to the stringent assumptions of the Rasch model, an appropriate method of analysis as it focuses on modelling responses at the item rather than the test level (Kolen & Brennan, 1995; Wright, 1977). Use of the Rasch model in test development allows the eventual development of a bank of items calibrated on a common scale, any subset of which could potentially form a test to estimate an individual's ability. Once such a calibrated bank is achieved a much larger number of possible tests can be created. In particular, a computer adaptive testing approach might also be possible.

Some limitations of the present work should also be noted. For the purposes of Rasch analysis the sample size was relatively small and, at very least, an independent replication on a new sample of trainees is required. The cut points identified should therefore be viewed as preliminary. Considerably more data on clinicians independently judged to be competent are needed to establish definitive cut points. The practical obstacles to the obtaining of such data are considerable.

While we have argued that applied knowledge is necessary for a therapist to be competent in a particular psychological treatment, it is not sufficient. The assessment of applied knowledge almost certainly needs to be supplemented with an assessment of therapist skill in implementing the treatment. We have therefore developed, and are currently evaluating, a role play-based measure for use with simulated patients. The relationship between scores on the eMeasure and scores on this skill-based measure needs to be determined to investigate their relative contributions to the assessment of therapist competence.

## Conflict of interest

There are no other potential conflicts of interest.

## Figures and Tables

**Table 1:** Blueprint for CBT-E knowledge measure.

Content area/topic	% of Items		
	All items	Application & problem solving items	Theoretical knowledge items
Assessment and preparation for treatment	5	0	5
CBT-E stage 1	45	30	15
CBT-E stages 2 & 3	45	35	10
CBT-E stage 4	5	0	5

Total	100	65	35

**Table 2:** Model fit for sets of tests of varying length.

Test set	Test items (per individual test)		
	S-22	S-24	S-25
Number of unique items	16	16	17
Number of common items	6	8	8
Fit statistic (item–trait interaction)	311.01, *p* = 0.688	370.1, *p* = 0.097	456.1, *p* = 0.0002
Item location, mean (SD)	0.00 (1.18)	0.00 (1.46)	0.00 (1.43)
Item fit residual, mean (SD)	−0.11 (0.88)	−0.12 (0.97)	−0.10 (1.07)
Person location, mean (SD)	−0.98 (1.31)	−1.14 (1.31)	−1.09 (1.28)
Person fit residual, mean (SD)	−0.14 (0.74)	−0.13 (0.60)	−0.12 (0.60)
Misfitting items	0	1 × *p* < 0.01	3 × *p* < 0.01, 1 × *p* < 0.001

## Footnotes

1 Dr Danette McKinley of The Foundation for Advancement of International Medical Education and Research (Faimer).

2 Intelligent Assessment Technology www.intelligentassessment.com.
