# Supplementary material for: The development of an online measure of therapist competence
Source: Behav Res Ther. 2015 Jan;64:43–8. doi: 10.1016/j.brat.2014.11.007 (PMC4289913; doi:10.1016/j.brat.2014.11.007)
Supplement: Supplementary file 1 [file mmc1.docx]

| A patient returns to session 1 not having completed any records as requested. She explains that she tries not to think about eating at all during the day and that she does not like the idea of focusing on eating and binge eating. She adds that she can remember what she has eaten each day.  *Please assume you have already praised the patient for her efforts and acknowledged her difficulties. How would you most appropriately address the issue raised?*     1. Suggest experimenting with recording for a bit longer and then stopping if pre-occupation is still an issue. 2. Discuss again why recording is important, suggesting that your previous explanation may not have been clear. 3. Explain that recording of eating is essential and insist that it is done as a condition of continuing in treatment. 4. Express surprise that the patient has experienced such difficulties with recording and address motivation to change. 5. Explain that it is vital to record eating as it allows the therapist to see the details of the patient's eating habits. |
| --- |
| A patient has done well in treatment and has not binged for 3 weeks. She attends session 17 very distressed because she has had a large binge the day before which was accompanied by vomiting. She feels very discouraged as she was doing very well and she does not know why the binge happened. She is now feeling that she is back to square one. She had thought that she was “over the eating disorder” and that it was possible to live her life without the eating disorder intruding.  *What is the most appropriate way to help her understand what has happened?*   1. Explore the influence of her dietary rules 2. Investigate the role of her regular eating over the last few days 3. Conduct a historical review with the patient 4. Explain the notion of mindsets 5. Examine her beliefs about the importance of weight and shape |
