# Supplementary material for: The development of an online measure of therapist competence
Source: Behav Res Ther. 2015 Jan;64:43–8. doi: 10.1016/j.brat.2014.11.007 (PMC4289913; doi:10.1016/j.brat.2014.11.007)
Supplement: Supplementary file 2 [file mmc2.docx]

You have introduced problem solving with a patient.  She returns to the next session having completed the exercise, as illustrated below.  After praising her for her efforts you agree to discuss how she managed the exercise and ways she could improve her problem solving.


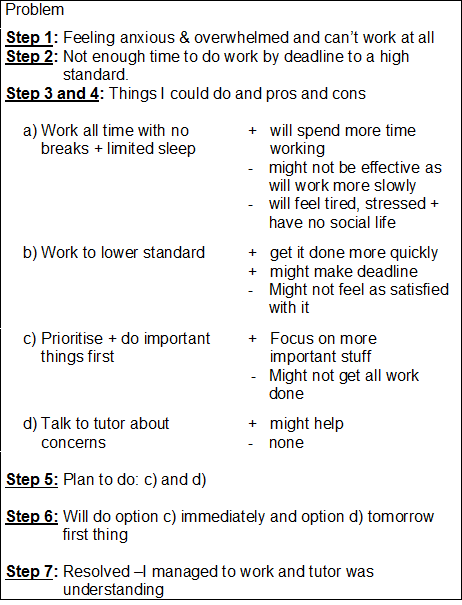


[**Click to view full size**](javascript:imagePopUp('image1411401465428340136',%20462,%20605))

***What is the best way to improve her problem solving?***

1. Step 1 or 2 should be linked to a change in her eating.
2. The problem should be more clearly specified in Step 1 and 2.
3. It would be better to pick just one clear option in Step 5 rather than two.
4. It would be better to concentrate on the problematic thinking style rather than a particular problem.
5. It would be better to focus on the acquisition of skills in Step 7 rather than on the particular solution.
6. It would be better to focus on challenging beliefs about high standards in option b).
