# Supplementary material for: The development of an online measure of therapist competence
Source: Behav Res Ther. 2015 Jan;64:43–8. doi: 10.1016/j.brat.2014.11.007 (PMC4289913; doi:10.1016/j.brat.2014.11.007)
Supplement: Supplementary file 3 [file mmc3.docx]

***What is the most important reason for addressing patients’ frequent body checking?***

1. It maintains the over-evaluation of shape and weight by providing unreliable information.
2. It maintains an avoidance of weighing by providing an alternative form of information about weight and shape.
3. It maintains pre-occupation with thoughts of weight and shape.
4. It maintains the belief that patients are fat by increasing their negative thoughts about their bodies.
5. It maintains dieting by providing information that patients are not “in control”.
